# Association of Helicobacter Pylori in Carcinoma Stomach at Maharaja Krishna Chandra Gajapat Medical College: A Prospective Study

**DOI:** 10.7759/cureus.30709

**Published:** 2022-10-26

**Authors:** Amar Kumar Behera, Manasa Ranjan Dash, Dhirendra Nath Soren, Kedar Nath Nayak, Deba Prasad Rath, Sudhansu Behera

**Affiliations:** 1 Department of General Surgery, Government Medical College & Hospital, Sundargarh, IND; 2 Department of Pediatric Surgery, Maharaja Krishna Chandra Gajapati Medical College & Hospital, Berhampur, IND; 3 General Surgery, Veer Surendra Sai Institute of Medical Sciences and Research, Burla, IND; 4 Department of General Surgery, Maharaja Krishna Chandra Gajapati Medical College & Hospital, Berhampur, IND

**Keywords:** gastrointestinal endoscopy, stomach carcinogenesis, endoscopic biopsy, h&e staining, histopathology examination, rapid urease test, helicobacter pylori, carcinoma stomach

## Abstract

Introduction

Gastric cancer is the fourth most common type of cancer and the second leading cause of cancer-related death in the world. The etiology of gastric cancer includes *Helicobacter pylori *infection, diet, lifestyle, tobacco, alcohol, and genetic susceptibility. Upper gastrointestinal endoscopy (UGIE) is the most effective method for examining the upper gastrointestinal tract as compared to the other examination tools.

Objective

To study the histopathological finding of upper gastrointestinal endoscopic biopsies and its association with *H. pylori* in cases of carcinoma stomach.

Materials and methods

This was a hospital-based observational study carried out in the Department of Surgery, at Maharaja Krushna Chandra Gajapati Medical College, Berhampur, a tertiary care hospital in Eastern India. Study population consisted of 106 patients for a period of 2 years from July 2019 to June 2021, after due consideration of the inclusion and exclusion criteria. Endoscopic location and pathological types of the gastric lesion were noted, and all biopsy specimens were investigated to see the presence of *H. pylori* by rapid urease test (RUT) and histological examination in the form of Giemsa and H&E stain.

Results

In the present study of 106 cases, 62 cases (58.49%) were found to be positive for *H. Pylori* by RUT and 72 cases (67.92%) were positive for *H. pylori* by smear staining. In histopathological study, 72 cases (67.92%) were of intestinal type of carcinoma and 34 cases (32.07%) were of diffuse type of carcinoma. Smear for *H. pylori* was positive in 56 cases (77.78%) among the 72 cases of intestinal type of carcinoma stomach. Whereas only 16 cases (47.05%) were found to be smear-positive for *H. pylori* among the 34 cases of diffuse type of lesion. Irrespective of histological type, *H. pylori* was positive in 67.92% of patients with carcinoma stomach. This association was statistically significant (p<0.001) and indicates its role in intestinal type of gastric carcinoma.

Conclusion

There is a high frequency of *H. pylori* infection in cases of stomach cancer. This study confirmed the higher association of *H. pylori* infection with gastric cancer. Its association with the intestinal histological variety of stomach cancer is more common than diffuse type. The prevalence of *H. pylori* infection in distal stomach carcinoma is higher than proximal.

## Introduction

The incidence of gastric cancer varies in different parts of the world and among different ethnic groups [1]. As per the Indian Council of Medical Research (ICMR) 2014, Consensus Document for Management of Gastric Cancer, prepared as an outcome of the ICMR Subcommittee on Gastric Cancer, India has a low incidence (approximately 34000) of gastric cancer as compared to that in Western countries [2]. The geographical distribution and etiology of gastric cancer differ widely in different geographical regions [3].

*Helicobacter pylori* is a gram-negative microaerophilic bacterium that resides in the mucous lining of the stomach. Warren and Marshall first demonstrated *H. pylori* infection in the development of gastrointestinal disease, for which they won the Nobel Prize [4]. *H. pylori* is thought to cause chronic gastritis, peptic ulcer disease, gastric carcinoma, and mucosa-associated lymphoid tissue (MALT) lymphoma [5]. *H. pylori* has been labeled as level I carcinogen [3]. *H. pylori* is considered as the main cause of chronic atrophic gastritis and thus may play a role in gastric carcinogenic process [6].

Several diagnostic methods utilizing invasive or non-invasive techniques are developed to detect *H. pylori* infection [7]. The invasive technique uses an upper gastrointestinal endoscopy (UGIE) for obtaining multiple biopsies from different sites of the stomach for culture, histological examination, RUT, PCR, etc. While non-invasive tests include urea breath test, stool antigen test, and blood serology [8]. UGIE is the most effective method for examining the upper gastrointestinal tract as compared to the other examination tools such as an upper GI barium series, computed tomography (CT), and magnetic resonance imaging (MRI) [9]. Seroprevalence of *H. pylori* infection in adult populations of India, Bangladesh, Pakistan, and Thailand varies from 55% to 92% whereas in China and Japan remains at 44% and 55%, respectively [10]. This study was conducted to know histopathological findings of upper gastrointestinal endoscopic biopsies and its association with *H. pylori* in cases of carcinoma stomach with insight into its symptoms and signs.

There have been various papers showing the association between *H. pylori* infection and gastric cancer. However, such studies are still lacking in the developing world, where the incidence of *H. pylori* is thought to be still on the rise. This study was done to find the association in region of interest, i.e., the western part of Odisha state.

## Materials and methods

This was a hospital-based prospective observational study carried out in the Department of Surgery at Maharaja Krushna Chandra Gajapati Medical College, Berhampur. Total 106 patients of both sexes and of all age groups who were admitted over a period of 2 years from July 2019 to June 2021 including 6 months of follow-up were included in this study by simple random sampling after inclusion and exclusion criteria applied in different categories. The study protocol was reviewed and approved by the Institutional Ethical Committee (I.E.C. No.: 979) of MKCG Medical College and Hospital, Berhampur. A detailed history and clinical examination were done on each patient according to protocol.

Inclusion criteria were all patients who were histopathologically confirmed to have carcinoma stomach. The study excluded patients who were on anti-*H. pylori* treatment within 6 months, cases who came negative for carcinoma stomach on histopathological study even if positive for *H. pylori*, and patients refusing/not willing to give consent for the study.

Methodology

A prospective study of the patient who presented with upper GI symptoms and suspected to have carcinoma stomach was investigated in the form of ultrasound, CT scan, and UGIE. On endoscopy, in patients who had suspicious lesion in stomach wall, four to six biopsies were taken from and around the growth as per international protocol. Also, from tissues away from the suspected tumor as there is a chance of proximal migration of *H. pylori* for those on proton pump inhibitor (PPI). So, biopsy from uninvolved corpus was also taken to confirm *H. pylori* on histopathology. All patients from whom biopsies were taken underwent RUT using PYLO DRYTM kit (Halifax Research Laboratory, Kolkata, India) as per its user manual. After each biopsy, the biopsy forceps were sterilized. All the biopsy specimens were subjected to both histopathological study and smear for *H. pylori*. We classified the carcinoma as per Lauren’s classification. According to Lauren’s criteria, gastric cancer is classified into two main types: intestinal and diffuse type. The stomach was divided into two regions proximal (cardia, fundus, and body) and distal (antrum and pylorus) for further stratifying the *H. pylori* as per the location.

All the patients in this study group underwent UGIE. The procedure was performed in overnight fasting state. The UGIE was conducted with Olympus, a flexible fiber-optic endoscope with the patient lying in left lateral positions. The stomach was looked for any inflammatory lesion, ulcer, or growth. Endoscopic biopsies were taken from the abnormal-looking area, growth, and the edge of the ulcer crater depending on the findings. A biopsy of gastric tissue is placed into a medium containing urea and a pH indicator. When the bacterial urease splits the urea, the liberated ammonia will increase the pH; this is recognized by a color change in the test indicator.

Biopsy specimens were sent to the pathology department in formalin solution for histopathological study. The smears were stained with modified Giemsa and Gram Stain and seen under an oil immersion lens to identify, the organism by its pathognomic “S” shape, gram-negative, and blue color in Giemsa stain. *H. pylori* positivity status was determined in each patient. All patients provided written informed consent.

We utilized IBM SPSS Statistics for Windows, Version 24.0. (IBM Corp., Armonk, NY) for analyzing the data. Calculated p-value using Chi-square test. We considered p value < 0.05 as statistically significant.

## Results

Out of 106 cases included in this study, most of the patients were between 51 and 60 years of age (39.6%) with mean age and SD of 51.4±3.8 years. The youngest patient in the study was 29 years old and the oldest was 75 years old. More than 60% of the total cases included in this study were found to be between 40 and 60 years of age. The mean age of our study group was 53.51 years. Out of 106 patients, 84 were males and 22 were females, showing male predominance (Table 1).

**Table 1:** Age and gender distribution

Age	Male (n=84)	Female (n=22)	Total (n=106)
(years)	Number (%)	Number (%)	Number (%)
21-30	02 (2.4%)	0 (0 %)	02 (1.9 %)
31-40	06 (7.1%)	02 (9.1%)	08 (7.6 %)
41-50	22 (26.2%)	06 (27.3%)	28 (26.4 %)
51-60	34 (40.5%)	08 (36.3%)	42 (39.6 %)
61-70	14 (16.7%)	04 (18.2%)	18 (16.9 %)
71-80	06 (7.1%)	02 (9.1%)	08 (7.6 %)

In the present series, the most common symptom was loss of appetite and/or loss of weight which were seen in 80 (75.5%) cases and the least common symptoms were dysphagia and hematemesis (Table 2).

**Table 2:** Incidence of different presenting symptoms

Symptoms	Number (%)
Loss of appetite/ loss of weight	80 (75.5 %)
Abdominal pain	76 (71.7 %)
Vomiting/nausea	68 (64.1 %)
Dyspepsia	44 (41.5 %)
Abdominal distension	38 (35.8 %)
Lump/Mass in abdomen	38 (35.8 %)
Melena	14 (13.2 %)
Dysphagia	02 (1.9 %)
Hematemesis	02 (1.9 %)

Anemia (Hb< 10 g%) was found in around 73.6% of the patients. Cachexia (BMI < 20 kg/m2) was seen in 41.5% of the cases. The palpable epigastric lump was present in 35.8% of cases and 20.8% of the cases showed upper abdominal tenderness. Peritoneal metastasis causing ascites was seen in 28.3% cases. Other clinical signs were epigastric tenderness, dependent edema, and Virchow’s node (Table 3).

**Table 3:** Physical signs

Signs	Number (%)
Anemia (Hb< 10 g%)	78 (73.6 %)
Cachexia (BMI < 20 kg/m^2^)	44 (41.5 %)
Palpable epigastric mass	38 (35.8 %)
Ascites	30 (28.3 %)
Epigastric tenderness	22 (20.8 %)
Dependent edema	10 (9.4 %)
Virchow’s node	02 (1.9 %)

Out of the total 106 cases of the study population, when UGIE biopsy specimens were subjected to histopathological study, 72 cases (67.92%) were found to be having the intestinal type of carcinoma stomach and 34 cases (32.07%) were found to be having diffuse type of carcinoma stomach (Table 4).

**Table 4:** Histological types of carcinoma stomach (according to Lauren’s)

Histological types carcinoma stomach	Number (%)
Diffuse type	34 (32.08 %)
Intestinal Type	72 (67.92 %)

According to location, out of 76 cases of distal carcinoma stomach, *H. pylori* positivity was seen in 58 (76.31%) cases, whereas in the rest of the 30 cases, the lesion involved proximal part of the stomach, *H. pylori* positivity was seen in only 14 (46.66%) cases (Table 5).

**Table 5:** H. pylori infection and predominant site of lesion

Site	Number (n=106)	*Helicobacter pylori*-positive number (%) (n=72)	*H. pylori*-negative number (%) (n=34)
Antrum/Pylorus	76	58 (76.31%)	18 (23.68%)
Body	16	10 (62.5%)	6 (37.5%)
Cardia	6	2 (33.33%)	4 (66.66%)
Fundus	8	2 (25%)	6 (75%)

On RUT, out of 106 cases of carcinoma stomach 62 cases (58.49%) were positive for *H. pylori*. Out of 62 cases of RUT-positive patients, 16 cases (47.05%) were of diffuse type and 46 cases (63.88%) were of intestinal type. On smear test for *H. pylori*, out of 106 cases of carcinoma stomach, only 72 cases (67.92%) were positive for *H. pylori*. Out of 72 cases of smear-positive patients 16 cases (47.05%) were of diffuse type and 56 cases (77.77%) were of intestinal type. On smear test, *H. pylori* incidence was more seen in the intestinal type (77.77%) than diffuse type (47.05%). It indicates higher *H. pylori* positivity in intestinal type of tumor for both RUT and smear test (p<0.001). Not much difference was found on RUT. Overall, the smear positivity for *H. pylori* in carcinoma stomach was 62.26% irrespective of the histological type of cancer (Table 6).

**Table 6:** Histological types of cancer along with smear and RUT positivity rate RUT: rapid urease test

Histological types	No. of Patients	Smear positivity number (%)	RUT positivity number (%)	P value	
				p<0.001	
Diffuse	34	16 (47.05%)	16 (47.05%)		
Intestinal	72	56 (77.77 %)	46 (63.88%)		

## Discussion

A total number of 106 cases were included in this study of all ages and sex. Most of the patients were between 51 and 60 years of age (39.6%). In the present study, the majority of cases occurred after 40 years of age. This finding was similar to the median age of 57 years noted by Halder et al. [11] who also observed that the incidence of gastric carcinoma in patients younger than 50 years was more common here than in the Western world. In this study, highest incidence of gastric cancer (39.6%) was found in the 51-60 years age group. More than 60% of the total cases included in this study were found to be between 40 and 60 years of age.

Out of 106 patients, 84 were males and 22 were females showing male predominance with a ratio of 3.8:1. A ratio of 2:1 (male: female) was found by Yeole [12] in 2008 in India. Loss of appetite and weight were the most common (75.5%) presenting symptoms.

Pain in the abdomen was a presenting complaint in 71.7% of cases. Various sources have noted varying percentages of patients with gastric cancer presenting with abdominal pain and the possibility of the description of a significant and bothersome discomfort as pain by the patient cannot be excluded. Wanebo et al. [13] state that abdomen pain was seen in over 50% cases. Stupart et al. [14] noted vomiting in 56 out of 67 cases studied (84%).

Vomiting of blood was a symptom in two cases and passage of black stool was complained by 13.2% of cases because of an ulcerative growth. This is comparable to the findings of Stupart et al. who reported hematemesis and/or melena in 19% of the cases [14].

One patient out of the 106 cases presented with dysphagia. Dysphagia was seen in 26% of cases by Wanebo et al. [13]. Dysphagia is a prominent clinical feature in cancers of the gastroesophageal junction and in some cancers of the gastric cardia. Pallor was a finding on physical examination in around 73.6% of the patients. Anemia is thought to be due to a combination of inadequate dietary intake, chronic blood loss, and deficiency of intrinsic factor. Anemia was one of the most common presenting findings by Halder et al. who noted it in 41% of the patients [11].

In the present study out of 106 study population of carcinoma stomach, 34 (32.08%) cases were found to be diffuse type and 72 (67.92%) cases were of intestinal type. This is comparable to a prospective controlled study that enrolled 56 patients from the Federal University of Rio Grande do Norte, Natal, RN, Brazil [15] in which 34 tumors (60.7%) were intestinal type and 22 (39.3%) were diffuse type carcinoma stomach.

In this study, we compared *H. pylori* infection in different anatomical locations of the stomach. According to location, we found that distal gastric cancer is more associated with *H. pylori* infection, out of 76 cases of distal carcinoma stomach, *H. pylori* positivity was seen in 58 (76.31%) cases, whereas in the rest of the 30 cases lesion involved other part of the stomach, *H. pylori* positivity was seen in only 14 (46.66%) cases. This result is comparable to study done by Bhasin et al., [16] where out of total 80 cases of carcinoma stomach, 48 had intestinal type, 28 diffuse type, and four mixed type of carcinoma stomach were identified. Tumor located in the antrum were 37, in body 31, and at other sites 12 cases were recorded. Association of *H. pylori* infection is more with intestinal type and distally located tumor.

As per this study, 62 (58.49%) out of 106 cases were found to be positive for *H. pylori* by RUT and 72 (67.92%) cases were positive for *H. pylori* on smear with staining. This is similar to the findings of Thapa et al. [17] who found RUT positivity in 67.5% cases.

In our study, when examined by Giemsa smear and urease test, *H. pylori* positivity rate in tumor tissue of intestinal-type carcinomas was higher than that of diffuse carcinomas. A descriptive study was carried out at the Ziauddin Medical University, Karachi [18], and histological evaluations of 50 cases of carcinoma stomach were done, *H. pylori* were identified in 35 (70%) cases. Out of that, intestinal types were found in 30 cases, whereas 15 cases were of diffuse type.

In the present study, *H. pylori* infection was compared between intestinal and diffuse histological types of carcinoma stomach. *H. pylori* positivity rate in tumor tissue of intestinal-type carcinomas was higher than that of diffuse carcinomas. Irrespective of the histological type of lesion, 67.92% of gastric carcinoma are associated with *H. pylori* similar to the findings of 69% in Thapa et al. [17]. This is at par with the finding of 74% of Buruk et al. [19]. *H. pylori* infection rate in the intestinal type is 77.78% which is similar to that in the Buruk series. This association is statistically significant (p<0.001) and indicates its role in gastric carcinoma.

The limitations of our study are a single-center study, with a relatively small sample size. To know the exact prevalence, we need to carry out a multicenter study and employ a combination of other invasive and non-invasive investigations with higher sensitivity and specificity in a large number of patients.

## Conclusions

Carcinoma stomach has a male preponderance and most commonly presents in the sixth decade of life. This study confirmed the higher association of *H. pylori* infection with gastric cancer. Association with *H. pylori* infection is higher in developing countries and early detection of infection and prompt therapy for eradication will probably reduce the incidence of gastric cancer and its health-related burden in these countries. But to know the accurate incidence we need to find out and employ other possible investigations with higher sensitivity and specificity in a large volume of patients.

We observed a significant association between *H. pylori* infection and intestinal type of carcinoma stomach. As per anatomical location, the incidence and relative risk of *H. pylori* infection were significantly high in distal gastric cancer patients. So, in all locations *H. pylori* infection could be the etiological agent. Proper public awareness, the identification of transmission routes, and successful eradication of treatment will be the mainstay of future prevention of gastric carcinogenesis caused by *H. pylori*.
